# Endolysosomal vesicles at the center of B cell activation

**DOI:** 10.1083/jcb.202307047

**Published:** 2024-02-02

**Authors:** Saara Hämälistö, Felipe Del Valle Batalla, María Isabel Yuseff, Pieta K. Mattila

**Affiliations:** 1 Institute of Biomedicine, and MediCity Research Laboratories, University of Turku, Turku, Finland; 2 Turku Bioscience, University of Turku and Åbo Akademi University, Turku, Finland; 3 InFLAMES Research Flagship, University of Turku, Turku, Finland; 4 Cancer Research Unit and FICAN West Cancer Centre Laboratory, Turku, Finland; 5 Laboratory of Immune Cell Biology, Department of Cellular and Molecular Biology, Pontificia Universidad Católica de Chile, Santiago, Chile

## Abstract

The endolysosomal system specializes in degrading cellular components and is crucial to maintaining homeostasis and adapting rapidly to metabolic and environmental cues. Cells of the immune system exploit this network to process antigens or promote cell death by secreting lysosome-related vesicles. In B lymphocytes, lysosomes are harnessed to facilitate the extraction of antigens and to promote their processing into peptides for presentation to T cells, critical steps to mount protective high-affinity antibody responses. Intriguingly, lysosomal vesicles are now considered important signaling units within cells and also display secretory functions by releasing their content to the extracellular space. In this review, we focus on how B cells use pathways involved in the intracellular trafficking, secretion, and function of endolysosomes to promote adaptive immune responses. A basic understanding of such mechanisms poses an interesting frontier for the development of therapeutic strategies in the context of cancer and autoimmune diseases.

## Teaser

Hämälistö et al. review how B lymphocytes have adopted specialized ways to use endolysosomal vesicles to promote adaptive immune responses.

## Introduction

B cells fill a key position in the adaptive immune system: they are the only cells capable of producing high-affinity antibodies against invading pathogens (Batista and Harwood, 2009). During their maturation, B cells migrate from the bone marrow to secondary lymphoid organs, where they await activation by antigens (Ag). Via their unique B cell antigen receptor (BCR), B cells recognize Ag either as soluble structures, typically immune complexes, or displayed on the surface of antigen-presenting cells (APCs) such as macrophages or dendritic cells, which triggers the formation of a specialized cell–cell contact called an immune synapse (IS) (Kuokkanen et al., 2015; Spillane and Tolar, 2018; Yuseff et al., 2013). Engagement of the BCR triggers receptor-mediated Ag internalization into endolysosomal compartments where they undergo processing into peptides that are loaded on major histocompatibility class II complexes (MHC-II). These complexes are transported to the cell surface and are recognized by T-helper cells, critical orchestrators of adaptive immune responses (Roche and Furuta, 2015). The presentation of antigenic peptides coupled to MHC-II not only activates cognate T-helper cells but they also, in exchange, provide B cells with signals for survival and differentiation, leading to the generation of antibody-producing plasma cells. Various specialized endolysosomal vesicles play a key role not only in Ag processing but also, e.g, in Ag extraction, required for extracellular detethering of Ag to enable its internalization and further processing, which is facilitated by localized exocytosis at the IS between the B cell and the Ag-tethered surface, such as APC (Yuseff et al., 2011). Our understanding of the different vesicle pools, their roles, and connections remains particularly poor in lymphocytes, and studies are challenged by the small and dynamic nature of these cellular structures.

To maintain cellular homeostasis, the highly heterogeneous endolysosomal vesicles carry cargo between various cellular compartments or redirect internalized cargo to different destinations for degradation (Bonifacino and Neefjes, 2017). At the same time, lysosomes have important signaling functions, for example, coupled to cell death, e.g., via lysosome membrane permeabilization and hydrolase release (Holland et al., 2020). Lysosomes also play an important role in cell survival by regulating metabolic activities in cells, for instance, by the major nutrient-sensing mammalian target of rapamycin complex 1 (mTORC1) complex that is recruited to the lysosome membrane by Ragulator-RAG GTPase complex upon abundant nutrient levels where it then monitors nutrient homeostasis, e.g., by preventing catabolism and promoting protein synthesis (Sancak et al., 2010; Ballabio and Bonifacino, 2020; Holland et al., 2020; Lamming and Bar-Peled, 2019; Perera and Zoncu, 2016). This represents an active and interesting research field as there is much to be discovered regarding the breadth of the lysosomal heterogeneity and their roles in cellular signaling and communication with other organelles (Ballabio and Bonifacino, 2020). In addition to functioning as messengers inside the cell, increasing evidence describes active roles for endolysosomal vesicles also in the extracellular space. Lysosomal exocytosis and the above-mentioned function in Ag extraction with the help of localized lysosome release is one aspect of this, but lysosomes have also been suggested to provide an origin for a subtype of extracellular vesicles (EVs) (Mathieu et al., 2021), in addition to the major exosome fraction of EVs that originate from the intraluminal vesicles (ILVs) of late endosomes (LE) or multivesicular bodies (MVB) (reviewed in Buzas [2022]). However, in general, the role of EVs in intercellular communication, in the immune system or B cells particularly, remains largely enigmatic.

In this review, we will cover what is currently known about the functions of lysosomal vesicles in B cells and how they are involved in Ag extraction and processing during early B cell activation, along with their novel roles as signaling platforms related to cell homeostasis. We also discuss other emerging functions of both endolysosomal vesicles and the highly intriguing EVs.

### Lysosome structure and maturation

While lysosomes are generally defined as intracellular vesicles carrying degradative acid hydrolases, recent studies revealed they are a heterogeneous pool of vesicles that differ in their subcellular localization, acidity, content, and signaling function (Ballabio and Bonifacino, 2020; Yang and Wang, 2021). Lysosomes are found as hundreds of vesicles in all mammalian cells, and their size can range between ∼50 and 1,000 nm. Close to 200 different proteins have been identified on the lysosomal membrane with various structural or signaling roles (Ballabio and Bonifacino, 2020). Type 1 proteins, such as lysosomal associated membrane protein-1 and -2 (LAMP-1 and LAMP-2), CD63 (LAMP-3), and lysosomal integral membrane protein-2 (LIMP-2) are the most abundant, constituting ∼50% of all transmembrane lysosomal proteins (Eskelinen, 2006; Gao et al., 2017). Via the accessible cytosolic domains of the transmembrane protein complexes, lysosomes act as scaffolds for transport machinery, signaling proteins, and other effector molecules (Inpanathan and Botelho, 2019). Heavily glycosylated LAMP molecules form a luminal glycocalyx structure and counteract the degradative actions of lysosomal acid hydrolases, thus helping maintain the barrier function of the membrane. Interestingly, however, this robust shielding mechanism can be modulated according to altered physiological situations; in mitosis, the architecture changes so that the expression of lysosomal membrane proteins and proteolytic cathepsin enzymes are downregulated, the lysosomal pH increases, and the limiting membranes become more fragile without compromising the cell homeostasis, simultaneously allowing a regulated enzymatic release to the nuclear region via lysosomal membrane permeabilization (Hämälistö et al., 2020; Stahl-Meyer et al., 2022). This suggests that the lysosome regulation can respond to cell-intrinsic cues by tuning down protein expression and/or functions, in this case, to shield the exposed genomic content from degradative enzymes (Hämälistö et al., 2021).

In general, the expression levels of the most prominent lysosomal proteins are regulated by the transcription factor EB (TFEB) (Roczniak-Ferguson et al., 2012). Overexpression of TFEB promotes, e.g., the recruitment of lysosomes to the plasma membrane (PM) and Ca^2+^-driven lysosomal exocytosis by the activity of the lysosomal Ca^2+^ channel mucolipin receptor MCOLN1 (Medina et al., 2011). TFEB itself and its subcellular localization are regulated by various phosphorylation events. In its inactive state, TFEB is phosphorylated by mTORC1 on residues S142 and S211 and resides mainly in the cytosol and on the lysosomal membrane (Settembre et al., 2011, 2012; Roczniak-Ferguson et al., 2012; Napolitano et al., 2018). Upon starvation or cellular stress, mTORC1 is inhibited, leading to loss of TFEB phosphorylation and TFEB nuclear translocation; however, during starvation, phosphorylation by extracellular regulated kinase-1 (Erk-1) on TFEB S142 is reported (Napolitano et al., 2018; Settembre et al., 2011). In the nucleus, TFEB upregulates the expression of genes encoding lysosomal proteins (Roczniak-Ferguson et al., 2012). The nuclear export of TFEB is promoted by nutrient levels and regulated mTOR-dependent phosphorylation on TFEB S138 and S142 (Napolitano et al., 2018).

During the immune response, lymphocytes undergo enhanced proliferation stages where B cells simultaneously rearrange and mutate their immunoglobulin locus to increase the Ag affinity of the BCR, later to be secreted as antibodies (Liu et al., 2023). How lysosome biogenesis is coupled to B cell proliferation and maturation is not known.

**Figure 1.**
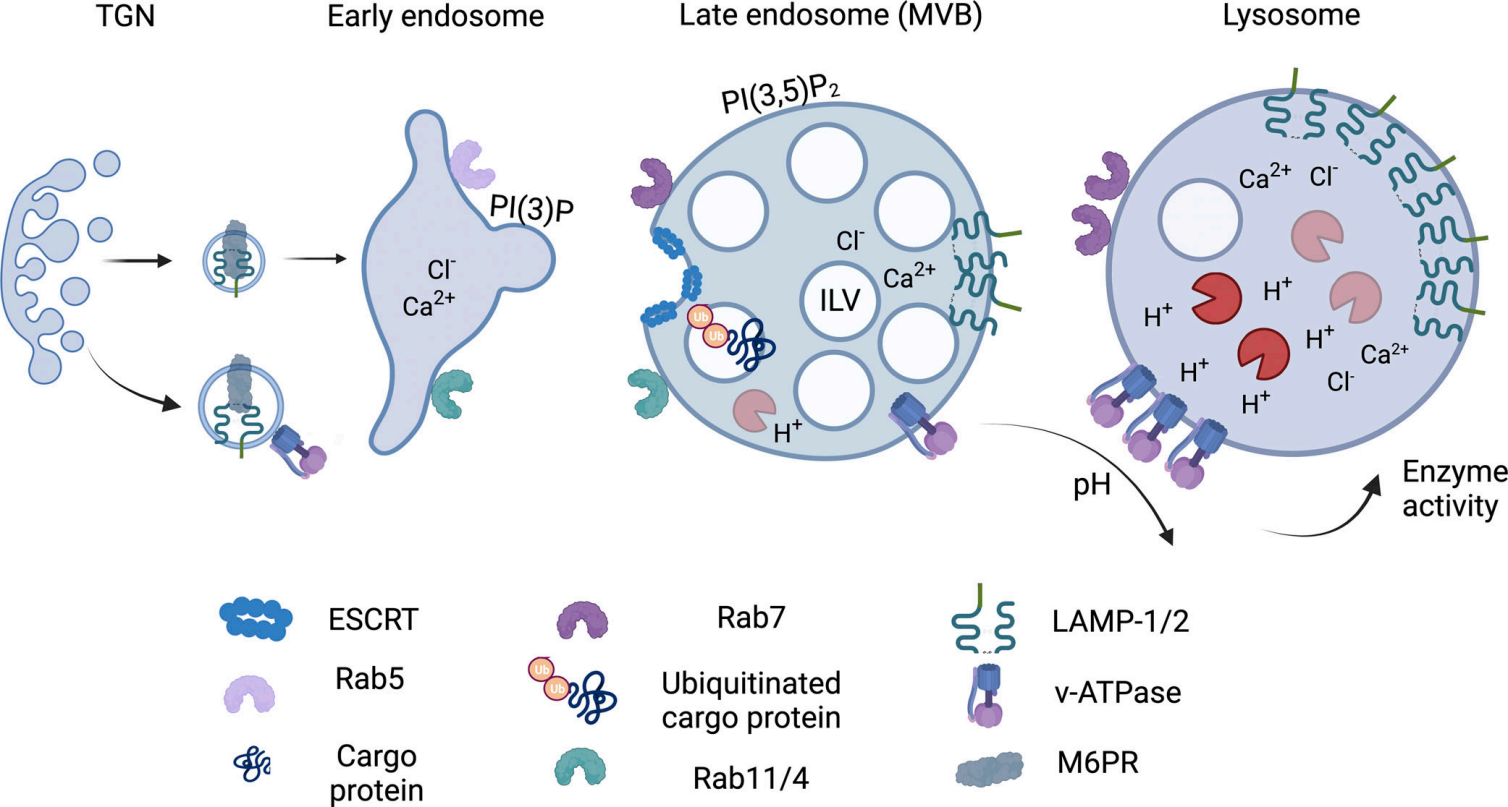
**An overview of endosome maturation.** During endosomal maturation, the EE GTPase Rab5 and lipid PI(3)P are replaced with Rab7 and lipid PI(3,5)P_2_, respectively, to trigger maturation into lLEs. The size of the endosomes increases and molecules such as LAMPs and the V-ATPase are transported from the trans-Golgi to the maturing endosomes via the mannose-6-phosphate receptor (M6PR). The V-ATPase, as the main H+ pump on the late endosomes and lysosomes, generates an acidic environment in the vesicle lumen. The membrane invagination process is driven by the timely delivery of the cytoplasmic ESCRT complex proteins to the limiting endosomal membrane (i.e., the membrane facing the cytoplasm) thereby creating ILVs inside MVBs or LEs. Here, the ubiquitinated membrane proteins stemming from endocytic or Golgi membranes are sorted into the growing closed ILVs. The ILVs can be released to the extracellular space as exosomes (a subtype of EVs) via MVB fusion with the plasma membrane, or the EE/MVB can generate Rab4/Rab11-rich recycling endosomes. The ion concentration during the maturation process steadily increases and the acidifying environment allows for the optimal enzyme activity inside the lysosomes.

The classical model of the endosomal maturation pathway, leading to the formation of conventional lysosomes, stems largely from studies in epithelial cell types (Fig. 1). Notably, endolysosomes are diverse, and their maturation pathways rely on the cell type as well as internalized cargo (Yang and Wang, 2021; Huotari and Helenius, 2011). The maturation of the endocytic vesicles toward late endosomes or lysosomes involves several steps; however, the timing and sequential order of events are not still entirely clear (Podinovskaia et al., 2021). The details of the classical model of endolysosomal maturation have been well-reviewed in the literature (Yang and Wang, 2021; Huotari and Helenius, 2011). Briefly, early endosomes (EE) mature into late endosomes (LE) upon switching the EE-specific Rab5 into LE-specific Rab7 (Rink et al., 2005), concurrent phosphatidylinositol (PI) conversion from PI-3-phosphate (PI(3)P) in EEs into PI-3,5-biphosphate PI(3,5)P_2_ in LEs. This occurs alongside the invagination of the EE membrane that forms ILVs leading to the formation of MVB or the late endosomal pool. This process of endosomal maturation involves dynamic and continuous rearrangements of the protein composition at the endosome limiting membranes, with the endosomal sorting complex required for transport (ESCRTs) proteins playing a key role (Hurley et al., 2020). The molecules destined for recycling endosomes are sorted with the help of ESCRT and retromer complex proteins in cooperation with various sorting nexins in the MVBs or at EEs and marked with Rab11 or Rab4 GTPase, respectively (Borchers et al., 2021; Chen et al., 2019). Most of the proteins with ubiquitin signals are sorted into ILVs in the invaginating EEs, and some of them are targeted back to PM (Piper et al., 2014). The MVB limiting membrane can be fused with the PM to induce the extracellular release of ILVs (as exosomes, a type of EVs), or it can fuse with an existing lysosome to be converted into a classical degradative lysosome.

Critically important for endolysosomal maturation is acidification, which gradually increases starting from the early endosomes (pH 5.9–6.8) to the late endosomes (pH 4.9–6.0) and lysosomes (pH 4.5–5) and is linked to the progression of the Rab5/7 switch (Hu et al., 2015; Podinovskaia et al., 2021). The acidic environment is generated by increasing the expression of the V-ATPase H+ pump on the endosomal membrane. From the trans-Golgi network, the transport of the V-ATPase is mediated by the lysosome-targeting mannose 6-phosphate receptor (M6PR) (Wang et al., 2020; Braulke and Bonifacino, 2009), where coordinated trafficking to and from lysosomes is crucial to maintain organelle fitness and a sufficient amount of molecules for nutrient recycling (Yang and Wang, 2021; Huotari and Helenius, 2011; Saftig and Klumperman, 2009).

The morphology of the endolysosomes undergoes major transformations during maturation. The EEs elongate with tubular extensions that are lost upon the maturation process, and the LEs and lysosomes acquire an oval-to-round morphology and grow in size. In this process, the recently described retromer-like retriever complex and the accompanying sorting nexin SNX17 are in a key position in mediating the transport of transmembrane proteins e.g., integrins from the endosome to the cell surface (reviewed in Chen et al. [2019]). Critical for endolysosome size regulation is the various lipid species e.g., the phosphoinositide phosphates (PIPs) that function as cues for the recruitment of various effector proteins to the lysosomes: too low or too high PI(3,5)P_2_ levels result in swelling and dysfunction of the endolysosomal structures (Ebner et al., 2019; Podinovskaia et al., 2021). Although vesicles at different stages may have similar protein markers and localization profiles, the end-stage, fully mature lysosomes are distinguished by their high hydrolase content and electron-dense appearance, which sets them apart from acidic late endosomes (Neiss, 1983; Tjelle et al., 1996).

### Lysosome positioning and function

Canonically, lysosomes are associated with actin and microtubule cytoskeleton and can be found distributed across the cell depending on the homeostatic conditions (Oyarzún et al., 2019). While short-distance endolysosomal vesicle mobility is typically achieved by local actin reorganization (Cordonnier et al., 2001), for long displacements, lysosomes move along the microtubule network via the action of motor proteins such as kinesins and dyneins and their adaptors (Roney et al., 2022; Gennerich and Vale, 2009; Ballabio and Bonifacino, 2020).

**Figure 2.**
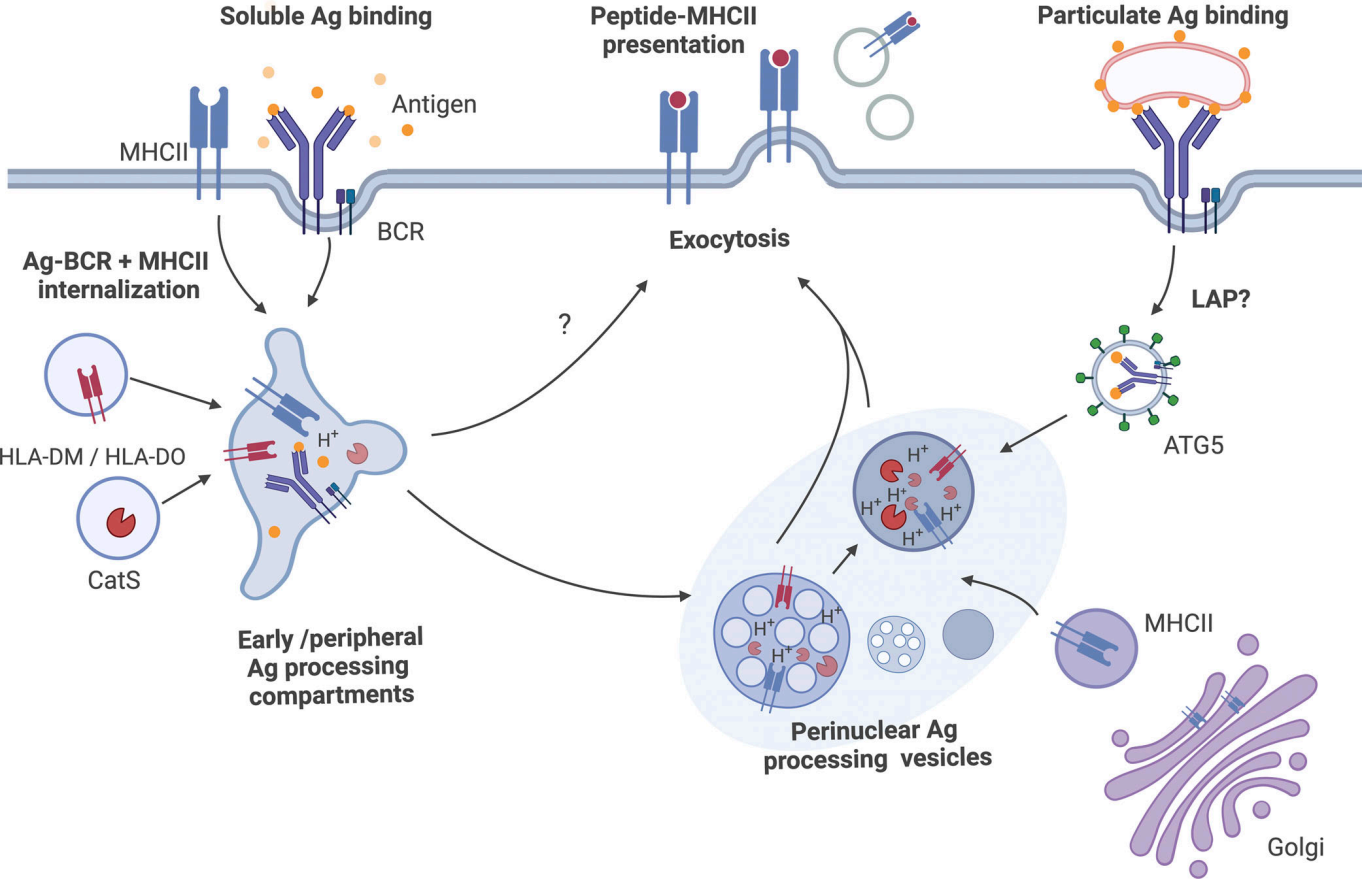
**Antigen processing pathways in B cells.** Perinuclearly located MHC-II^+^ vesicles of lysosomal or multivesicular nature are considered the main Ag processing compartments, where Ag-derived peptides, after digestion by cathepsins, are loaded on MHC-II in a process tightly regulated by molecules such as the HLA-DM and HLA-DO chaperones. Peptide-loaded MHC-II complexes are then directed for cell surface presentation via exocytosis or they can also be secreted as extracellular vesicles. Two specialized pathways have been highlighted as possible routes for targeting the BCR-bound Ag to the Ag processing vesicles. For soluble Ag, simultaneous targeting of Ag-BCR and plasma membrane-derived MHC-II to the peripherally located acidic early Ag processing vesicles has been shown (Hernández-Pérez, 2019). As these early vesicles already show the key features of Ag processing compartments, some peptide-loaded MHC-II could be directed to the cell surface already from this stage. Surface-bound Ag, on the other hand, has been proposed to enter an ATG5-dependent pathway, presumably involving a form of LC3-associated phagocytosis (LAP) (Arbogast et al., 2019). Overall, it is likely that the endolysosomes processing Ag form a heterogenous gradient with varying amounts of the protein components necessary for pMHC-II generation. The nature of these vesicular compartments is likely to depend on the status or type of the B cell and the Ag.

Interestingly, the subcellular positioning of lysosomes is also linked to their acidity and motility, where peripheral lysosomes have higher, less acidic pH than the ones residing perinuclearly (Johnson et al., 2016; Quick et al., 2023). Lysosomes in the perinuclear region exhibit also reduced mobility compared with those in the periphery. This diminished transport within the perinuclear domain may be due to variances in cytoskeletal connections within this region, an effect of progressive lysosomal maturation state, or underscore the emerging role of the endoplasmic reticulum–lysosome contact sites (Cabukusta and Neefjes, 2018; Rayens et al., 2022). Various signals or conditions affect lysosome positioning. For example, nutrient deprivation can trigger the lysosomes to accumulate in the perinuclear region, whereas a nutrient-rich state promotes peripheral localization (Ballabio and Bonifacino, 2020; Korolchuk et al., 2011). In B cells, the autophagic regulator autophagy related (ATG) 5, probably in a complex with ATG12 and ATG16-like 1, is required for efficient lysosome polarization to the IS and the internalization of synapse-tethered or particulate antigen to MHC-II vesicles, in a manner proposed to be reminiscent of LC3-associated phagocytosis (LAP) (Fig. 2; Arbogast et al., 2019). In line with this, another study reported the accumulation of LC3 with the internalized antigen (Runsala et al., 2023), together indicating that autophagy-linked protein machinery is activated in response to B cell IS formation and involved in targeting the internalized antigen for further processing. Interestingly, toll-like receptor ligands, such as lipopolysaccharide (LPS), restrict lysosome repositioning to the IS of B cells by triggering autophagy-dependent degradation of GEF-H1, a Rho GTP exchange factor required for stable lysosome recruitment at the synaptic membrane (Lagos et al., 2022). The BCR activation state can regulate the autophagic signature in a manner dependent on the cell stage and the local microenvironment: it was recently proposed that a switch from mTORC1-driven canonical to non-canonical autophagy occurs in the germinal center (GC) B cells upon B cell activation, proposing that the B cell stage and location in situ may influence the responses to antigen exposure via alternating the autophagy route (Martinez-Martin et al., 2017; Raza and Clarke, 2021). This switch might occur to cover the increased need for energy during B cell selection and differentiation and suggests that this process is finely tuned. Thus, sophisticated regulation of lysosomes and lysosome-related organelles (LROs) (detailed in the next chapter) in collaboration with autophagy machinery is emerging with implications for the overall immune response, although very little is known about the molecular mechanisms behind these regulatory pathways. Overall, this intricate regulation of autophagy together with lysosome positioning and dynamics in B cells opens doors for tuning immune responses with precision.

### Antigen processing compartments and other lysosome-related organelles

Endolysosomes within different immune cells display unique features. One key feature is the intracellular processing of extracellular-derived Ag for peptide-MHC-II (pMHC-II) presentation, a key to trigger the generation of both B and T effector cells responsible for the adaptive immune response. This process involves specialized lysosomal Ag processing compartments equipped with carefully controlled proteolytic enzymes. The general cascade of Ag processing for pMHC-II presentation has been intensively investigated and prominent reviews have been published on the topic (Roche and Furuta, 2015; Unanue et al., 2016; Watts, 2022).

While the vesicular identity and trafficking involved in Ag processing remain to be fully understood, the associated biochemical chain of events is relatively well characterized, mainly by studies in dendritic cells and macrophages. In brief, in the endoplasmic reticulum, the newly synthesized MHC-II complexes associate with the invariant chain (Ii), which protects the peptide-binding groove and provides targeting signals directing the complex to the Ag processing compartments. Reported to occur in the ILVs of the MBV/LEs, the sequential proteolytic processing of Ii is initiated and ultimately leads to a short CLIP peptide that remains to occupy the peptide-binding groove of MHC-II (Roche and Furuta, 2015). Finally, CLIP is removed by a chaperone HLA-DM (H2-M in mice), or HLA-DO (H2-O in mice) in mildly acidic compartments, allowing for Ag-derived peptide binding to the MHC-II and subsequent exit of the complex to the PM for peptide Ag presentation (Roche and Furuta, 2015; Watts, 2022). Processing of antigens occurs essentially by the same lysosomally enriched enzymes that process the Ii, the most prominent ones being asparaginyl endopeptidase and cathepsins, particularly Cathepsin S (Manoury et al., 2003; Shi et al., 1999). With their acidic and proteolytic environment, the Ag processing compartments show clear lysosomal character, yet their key role is to generate critical protein complexes for surface presentation rather than simply degrade cargo. Multivesicular and multilamellar compartments rich in MHC-II have been observed in B cells and dendritic cells by immunoelectron microscopy, reminiscent of Ag processing compartments positioning just before the terminal lysosomes on the endolysosomal roadmap (Peters et al., 1991; Kleijmeer et al., 2001).

How exactly the endolysosomal system is adopted to the specialized function of Ag processing and peptide loading remains unclear and is likely to alter between different cell types and stages. Interestingly, from the Ag processing machinery components, HLA-DO is most prominently expressed in B cells and is linked to tuning of the immunodominant peptide repertoire presented by B cells (Welsh and Sadegh-Nasseri, 2020). This is in line with the observed preference for HLA-DO activity, particularly in a mildly acidic compartment (Roche and Furuta, 2015), and the finding that BCR-mediated Ag processing occurs along the entire endosomal route, starting with the rapid fusion of internalized Ag into peripheral acidic compartments together with PM-derived MHC-II (Fig. 2; Hernández-Pérez, 2019). EE and LE markers often overlap in the Ag-containing compartments in B cells, further reflecting that the immune cell endolysosomal system has adapted hybrid forms to fulfill their specialized functions in Ag processing (Hernández-Pérez, 2019).

Ag processing compartments are considered LROs, specialized subcellular compartments, which share similarities with lysosomes, such as their low pH and hydrolase content, but exhibit distinct characteristics (Delevoye et al., 2019; Watts, 2022). LROs destined to undergo exocytosis are transported and tethered at specific domains of the plasma membrane (Kuppannan et al., 2022). This process is triggered by the activation of specific cell surface receptors, such as the T cell receptor or natural cytotoxicity receptors in natural killer (NK) cells. For instance, in cytotoxic T cells, LROs, termed lytic granules, harbor unique hydrolytic enzymes, perforin, and granzyme B; engage in polarized movement along microtubules; and then are released at the synaptic membrane after LRO fusion with the PM, promoting the elimination of target cells (Catalfamo and Henkart, 2003). Analogously, NK cells also secrete lytic granules, which contain granzymes A and B, FasL (also known as FasLG), granulysin, and IFNγ, to the synaptic membrane (Friedman et al., 2021). Receptor activation also results in Ca^2+^ signaling, which promotes the local exocytosis of LROs by SNARE proteins, including SNAP-23, VAMP2, VAMP7, syntaxin 3, syntaxin 4, and SCAMP (Blott and Griffiths, 2002), each exhibiting context-dependent roles in the orchestration of exocytotic events across diverse cell types. Ca^2+^ induction also promotes the transient engagement of Rab11 with lysosomes at the cellular periphery, facilitating lysosome exocytosis. Additionally, this process can be tuned by the physical association between Rab11 and the exocyst subunit Sec15 (Escrevente et al., 2021). Similarly, the exocyst complex was shown to promote lysosome tethering at the B cell synaptic membrane (Sáez et al., 2019); however, the role of Rab11 in lysosome secretion at the B cell synapse remains to be evaluated.

Another example is mast cells, where secretory granules, like those found in NK cells and platelets, release substances such as histamine and lysosomal hydrolases to initiate an inflammatory response (Azouz et al., 2014). Altogether, these results highlight a common mechanism involving microtubule-based transport, membrane trafficking, and fusion events at the immune synapse that are required for lymphocytes to acquire specific effector functions.

As the function of immune cells is to be sensitized by pathogenic products, it is not surprising that upon exposure, lysosomes are remodeled in different ways. Various vesicle-linked proteins have been shown to alter their intracellular positioning, for instance, upon BCR engagement (Music et al., 2022; Awoniyi et al., 2023). However, more efforts are required to dissect the functions of the different endosomal players in B cell activation. A proteomic analysis of isolated lysosomes shows how the exposure of different pathogens to murine macrophages alters the lysosomal protein expression profile in a manner specific to the type of pathogen (Gao et al., 2017). For example, the relative abundance of transmembrane proteins Lamp-1/Limp-2 or digestive hydrolases Cathepsin K or L was shown to be adjusted in a pathogen-specific manner, suggesting alternative regulatory mechanisms depending on the pathogen species and the nature of the external stimuli (Gao et al., 2017). Also, in macrophages and dendritic cells, activation by LPS drives microtubule-assisted lysosome tubulation and surface expression of MHC-II (Saric et al., 2016). In dendritic cells, the exclusive expression of Lamp-3 (or CD63) in the lysosomes and the cell surface promotes the delivery of pMHCII^+^ compartments to the PM as well as dendritic cell migration to draining lymph nodes (Liu et al., 2023). Whether Lamp1 and Lamp2, typically assumed to decorate the same vesicular compartments, also regulate differential endolysosomal functions, analogously to Lamp3, in immune cells, is not known.

### Lysosomes facilitate antigen extraction at the IS during B cell activation

**Figure 3.**
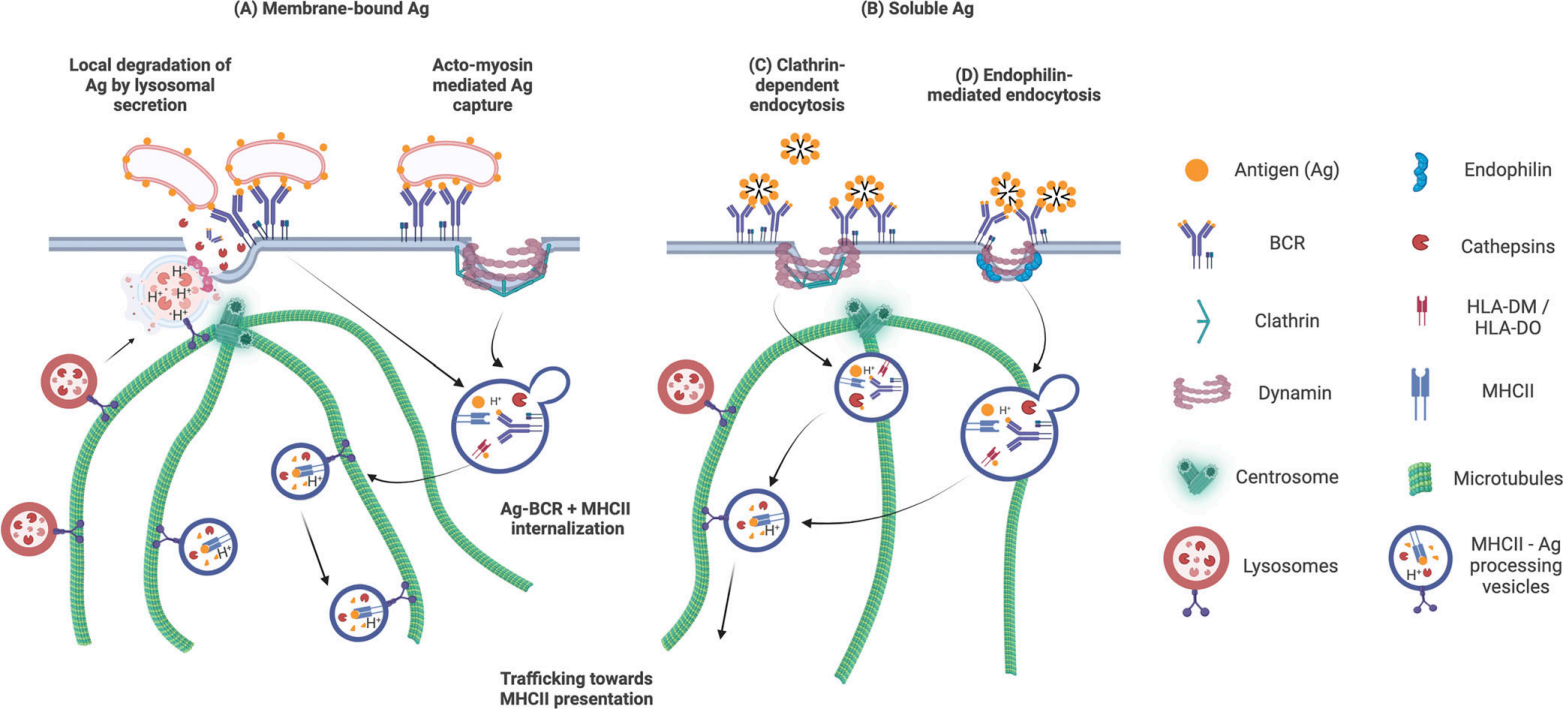
**Lysosome contribution to antigen extraction and internalization at the IS. (A)** When B cells encounter membrane-bound antigens, they rely on the balanced action of actin- and myosin-mediated forces and lysosomal exocytosis to extract and internalize the Ag and establish an IS. Lysosomes move on microtubule tracks directed by the MTOC and fuse with the plasma membrane. They secrete their acidic content to the synaptic cleft promoting further internalization and processing of antigens. **(B–D)** Soluble Ag (B) is internalized primarily by clathrin-mediated endocytosis (C). Alternatively, B cells employ fast endophilin-mediated endocytosis which can internalize both soluble or surface-tethered Ag (McShane and Malinova, 2022) (D). Within specific membrane patches primed by endophilin proteins, BCR-Ag binding leads to the induction of membrane curvature facilitated by endophilin proteins. Dynamin then promotes the scission of the curved membrane, resulting in the formation of an intracellular vesicle carrying the BCR-antigen complex. After internalization, all these vesicles subsequently fuse with late endosomes, enabling Ag processing and presentation to other immune cells.

In B cells, Ag recognition also boosts lysosome exocytosis, releasing digestive hydrolases to facilitate Ag extraction from antigen-presenting cell surfaces (Maeda et al., 2021). Here, cell signaling and Ag extraction are tightly coordinated in the synaptic structure formed between the B cell and Ag presenting surface (Batista and Harwood, 2009; Yuseff et al., 2011). During IS formation, B cells undergo rapid cytoskeletal remodeling, leading to changes in cellular morphology, and directed trafficking of polarity proteins and vesicular organelles to the IS to help extract the Ag (Kuokkanen et al., 2015). In addition to the lamellipodia-like actin-based spreading, myosin-dependent contraction helps accumulate the BCR-bound Ag at the center of the IS for internalization (Treanor et al., 2010; Bolger-Munro et al., 2019). The Ag-BCR microclusters form and accumulate in the middle of the IS within a couple of minutes upon Ag recognition (Bolger-Munro et al., 2019; Kuokkanen et al., 2015). Finally, the antigen is internalized via clathrin-mediated endocytosis or a recently reported fast endophilin-mediated route (Fig. 3; McShane and Malinova, 2022; Stoddart et al., 2002). In the case of B cell activation with soluble Ag, clathrin-mediated endocytosis is typically, but not exclusively, preferred (McShane and Malinova, 2022).

Lysosomes have a crucial role in facilitating antigen extraction at the IS to enable efficient B cell activation, but how are the lysosomes targeted to the IS? During the first steps of IS formation, the centrosome becomes detached from the perinuclear region in a process mediated by the depolymerization of actin and proteasome activity (Ibañez-Vega et al., 2019, 2021). Then, the centrosome is polarized to the Ag-BCR contact zone at the PM, guided by the activity of conserved polarity proteins, such as PAR3/aPKC/Cdc42 (Yuseff et al., 2013). The reorientation of the microtubule network enables the recruitment of lysosomes and other functional cellular compartments to the IS using molecular motors such as dyneins (Fig. 3; Reversat et al., 2015; Wang et al., 2017). After polarization to the IS, lysosomes fuse with the PM and secrete their acidic vesicle content to facilitate Ag extraction and presentation to T cells by, e.g., the hydrolase Cathepsin S (Yadati et al., 2020; Maeda et al., 2021; Yuseff and Lennon-Duménil, 2015). This Ca^2+^-regulated process, termed lysosomal exocytosis, was originally discovered to function in membrane repair (Reddy et al., 2001), and it can be detected by increased exposure of the luminal epitope of LAMP-1 at the extracellular side of the plasma membrane (Andrews, 2017). LAMP1^+^ endolysosomes in B cells express the V-SNARE VAMP-7 (also known as Ti-Vamp), which mediates fusion with the plasma membrane and lysosome exocytosis and thereby facilitates antigen extraction, processing, and presentation (Obino et al., 2017). Lysosome exocytosis in B cells has also been shown to involve permeabilization of the PM to facilitate efficient Ag extraction in a mechanism that requires BCR signaling and non-muscle myosin II activity (Maeda et al., 2021); in brief, the authors show that the higher the affinity of Ag to the BCR, the more permeable the PM becomes and the higher the rates of lysosomal exocytosis are, measured by increasing levels of LIMP-2 at the cell surface. Regarding B cell biology, the mechanisms triggering lysosomal exocytosis are not well described. For example, in epithelial-type cells, transcriptional response via TFEB is known to be a major contributor (Medina et al., 2011). However, considering the relatively fast (minutes, rather than hours) response from BCR-Ag binding to lysosomal exocytosis in B cells, it is reasonable to assume that the triggering does not require transcriptional changes, at least in early phases of B cell activation (Yuseff et al., 2011).

Another critical component during Ag extraction is the participation of the Exocyst complex, a hetero-octameric oligomer that functions as an anchoring component to target secretory vesicles to precise domains of the plasma membrane, thereby promoting their local secretion (Zeng et al., 2017). Indicative of the association of the exocyst complex in B cell IS, four of the eight exocyst subunits were found to be enriched in centrosome fractions isolated from activated B lymphocytes (Sáez et al., 2019). Upon activation, B cells upregulate acetylation and stabilization of their microtubule network in the vicinity of the MTOC. This releases the Exocyst subunit Exo70, and GEF-H1 from microtubules, which become associated with the synaptic membrane, enabling the successful docking of lysosomes at the IS. Cells silenced for Exo70 and GEF-H1 display defective Ag extraction, processing, and presentation (Sáez et al., 2019).

Thus, lysosomes have special roles in the IS and the subsequent B cell activation. However, more mechanistic insight on lysosome positioning and dynamics (fusion/fission) while establishing an effective IS in B cells remains to be discovered.

### Emerging roles of endolysosomal compartments in extracellular vesicle release and cellular signaling

In addition to the lysosome contents secreted for Ag extraction, B cells are an intense source of EVs, the destiny and diversity of which remain to be discovered (Kato et al., 2020; Muntasell et al., 2007; Buzas, 2022). Although EVs are often considered to originate from the late endosomal ILVs as exosomes or budded from the PM, they are also found to display lysosomal features, and lysosomes have been suggested to provide the origin for a subset of EVs (Mathieu et al., 2021). B cells have been shown to secrete Ag-presenting EVs containing lysosomal components such as LAMP-1, MHC-I, and MHC-II, and costimulatory molecules, which could induce Ag-specific CD4^+^ T cell response or cytotoxic CD8^+^ T cell response (Kato et al., 2020). EVs can mediate specific signaling functions in the immune microenvironment, making cell communication via EVs an intriguing area of research with the promise of novel therapeutic possibilities (Kugeratski and Kalluri, 2021; Buzas, 2022). For instance, EVs secreted by malignant B lymphoma cells Balm-3, Su-DHL-4, and OCI-Ly1 can shield the tumor cells from anti-CD20 antibody (rituximab)-mediated destruction via sequestering the antibodies on the EV-tethered CD20 (Aung et al., 2011). By silencing or inhibiting the LRO-associated ABCA3, which induces the production of EVs, the cells become more vulnerable to rituximab treatment (Aung et al., 2011). Furthermore, the Fas ligand expressed on B cell-derived exosomes induces apoptosis in CD4^+^ T cells (Klinker, 2014). Unlike soluble immunoglobulins released from the cells, the B cell EVs carry BCR immunoglobulins that can directly connect EVs to specific antigens or cells with immunoglobulin receptors, highlighting a different immunoregulatory mechanism for antibodies and immunoglobulin-bearing EVs (Gutknecht et al., 2023). On the other hand, EVs can alter the activation status of healthy donor B cells as shown by decreased global tyrosine phosphorylation and reduced proliferation (Rincón-Arévalo et al., 2022). Linkages between the endolysosomal system and the release of EVs are still very poorly understood, and more studies are needed to provide insights into which signals trigger the EV release and determine the cargo molecules that are loaded inside. The pH levels of the released EVs are not reported and, for instance, whether the B cell lysosomal hydrolase cathepsin S with optimal function at acidic pH (Manoury et al., 2003; Yadati et al., 2020) is recruited and retained functional inside the released EVs, or whether it is transported as non-functional unprocessed enzymes to target tissues, remains to be discovered. Elucidating this would add another layer of understanding the immune cell effects on intercellular communication. Notably, various B cell–specific surface markers highlight the potential of B cell–derived EVs for biomarker discovery for lymphoma or autoimmune conditions.

## Discussion and future perspectives

The small and crowded cytoplasmic space challenges the studies of the complex and specialized endolysosomal features of B cells (Hernández-Pérez and Mattila, 2022). Given this spatial restriction and high density of vesicles together with the highly specialized functional needs of B cells, it is not surprising that the organellar regulation would differ from, e.g., epithelial or neuronal type of cells, where the cytoplasmic space is larger and often more longitudinally organized. Interestingly, effective passive diffusion of small vesicular carriers has recently been reported (Sittewelle and Royle, 2023). Given the limited cytoplasmic space, together with the small size and heterogeneity of endolysosomal vesicles in B cells, the potential of passively diffusing vesicles should be considered in future research.

Profound aspects of B cell lysosomal biology are still unexplored. Some answers to the heterogeneity, atypical features, and specific functions could arise from a better understanding of the proteins linked to vesicle identity and trafficking, such as LAMP-1 and LAMP-2 as well as many different Rab GTPases. How are these key vesicle-linked proteins altered in response to antigenic or other stimuli? While it is well established that lysosomes are recruited to the IS during B cell activation, it is not known whether the lysosomes themselves are modified during this process. For example, it is tempting to speculate that Ag encounter and the following lysosomal exocytosis would involve lysosomal membrane permeabilization (Stahl-Meyer et al., 2022). What are the key factors distinguishing the Ag processing pathway from the canonical endolysosomal route, and how are these pathways coupled to lysosome exocytosis to facilitate Ag extraction? In line with the regulatory role of TFEB and its family member Transcription factor E3 (TFE3) in lysosomal exocytosis or autophagy in other cell models, TFEB and TFE3 have also been linked to adaptive immune responses and peptide-Ag presentation in dendritic cells, and diminished B cell activation has been reported in mice lacking TFE3 (Martina and Puertollano, 2017; Samie and Cresswell, 2015; Merrell et al., 1997; Brady et al., 2018). However, little is known about their mechanistic role in lysosome regulation in B cells. Identification of key molecules regulating the extended lysosomal functions in B cells would help unveil the orchestration of the peculiar mixed early and late endosomal vesicle repertoire in Ag processing (Hernández-Pérez, 2019) and the extracellular release of lysosomal components (Yuseff and Lennon-Duménil, 2015).

How do perturbations in lysosome homeostasis affect B cell activation? Insight can be gained by studying rare disorders such as lysosomal storage diseases (LSDs), where mutations in genes encoding for lysosomal hydrolases, transporters, or membrane proteins result in the abnormal accumulation of macromolecules within lysosomes leading to cell death and multiple organ dysfunction (Cabrera-Reyes et al., 2021). Most of the LSDs are associated with severe neurological defects; however, immune deficiencies and autoimmunity have also been observed (Rigante et al., 2017; Simonaro, 2016) and possibly often overlooked. Few studies have addressed how B cell activation is compromised in LSDs or when defective trafficking of lysosomal proteins occurs. For instance, mice deficient in the formation of M6P residues exhibit significant loss of cathepsin proteases in B cells, leading to lysosomal dysfunction with an accumulation of storage material, impaired Ag processing and presentation, and subsequent defects in B cell maturation and antibody production (Otomo et al., 2015). Peripheral B cells isolated from patients with Niemann-Pick disease type C1 (NP-C1), a rare lysosomal storage disorder resulting from mutations in an endolysosomal cholesterol transporter display increased levels of lysosomal glycosphingolipids (Lachmann et al., 2004) and B lymphocytes treated with U18666A, an inhibitor of the lysosomal cholesterol transporter, NPC1, accumulate lysosomes in the perinuclear region, suggesting that their function might be compromised.

The study of LSDs can therefore contribute to a better understanding of how lysosome fitness can impact B cell activation and humoral responses. The knowledge obtained from such severe pathological settings can reveal critical information that could be relevant for other conditions found in the general population, such as metabolic disorders including diabetes and obesity, where impaired lysosomal function is a common hallmark (Cabrera-Reyes et al., 2021).

More research is needed to understand the multifaceted functions of lysosomal vesicles in B cells. We expect that proteomics could be key in the quest to unravel important new insights into lysosome functions upon B cell activation. Analysis of isolated lysosomes, like in macrophages (Gao et al., 2017), or modern proteomics tools like LysoIP (Laqtom, 2023) and proximity proteomics (similar to Awoniyi et al. [2023]), combined, for example, with the information of the overall proteome recruited to the IS upon B cell activation (Cunha et al., 2023a *Preprint*, 2023b) hold great promise for new insights of the lysosomal heterogeneity and LRO functions. Understanding B cell activation at the molecular level would help us to decipher the pathogenesis of various autoimmune conditions or B cell lymphomas and has implications on immunotherapeutic aspects as well. Furthering the impact of this research even more is the role of B cells that has emerged in tumor immunology, where recent studies promote B cell–rich tertiary lymphoid structures (TLS) as a positive prognostic indicator (Trüb and Zippelius, 2021). Furthermore, EVs undoubtedly generate an interesting frontier with plenty of possibilities for future diagnostics or therapies, given their signaling functions, aided by specific surface markers that distinguish B cell–derived EVs from other sources.
